# The Outer Membrane Lipoprotein Tp0136 Stimulates Human Platelet Activation and Aggregation Through PAR1 to Enhance G_q_/G_i_ Signaling

**DOI:** 10.3389/fimmu.2022.818151

**Published:** 2022-02-28

**Authors:** Qiu-Yan Xu, Yong-Jing Wang, Li-Rong Lin, Li-Li Liu, Tian-Ci Yang

**Affiliations:** ^1^Center of Clinical Laboratory, Zhongshan Hospital of Xiamen University, School of Medicine, Xiamen University, Xiamen, China; ^2^Institute of Infectious Disease, School of Medicine, Xiamen University, Xiamen, China

**Keywords:** Tp0136, platelet activation, platelet aggregation, protease-activated receptor 1, G_q_, G_I_

## Abstract

**Background:**

Chancre self-healing, a typical clinical phenomenon of primary syphilis, is essentially wound healing. The first response to a wound is constriction of the injured blood vessels and activation of platelets to form a fibrin clot. However, the role of *Treponema pallidum* in platelet activation and clot formation remains unclear.

**Objectives:**

We aimed to elucidate the role of the outer membrane *Treponema pallidum* lipoprotein Tp0136 in human platelet activation and aggregation and explore the related mechanism.

**Methods:**

A series of experiments were performed to assess the effects of Tp0136 on human platelet activation and aggregation *in vitro*. The effect of Tp0136 on platelet receptors was studied by detecting PAR1 protein levels and studying related receptor sites. The involvement of the G_q_/G_i_ signaling pathway downstream of PAR1 was explored.

**Results:**

Tp0136 significantly accelerated the formation of human platelet clots as well as platelet adhesion to and diffusion on fibrinogen to promote platelet aggregation. Tp0136 also potentiated P-selectin expression and PF4 release to promote platelet activation and downregulated PAR1 expression. The activation and aggregation induced by Tp0136 were reverted by the specific PAR1 antagonist RWJ56110 and the human PAR1 antibody. In addition, Tp0136 significantly enhanced G_q_ and G_i_ signaling activation, thereby triggering p38 phosphorylation and Akt-PI3K activation, increasing the release of intraplatelet Ca^2+^ and attenuating the release of cytosolic cAMP. Furthermore, the specific PAR1 antagonist RWJ56110 significantly suppressed G_q_ and G_i_ signaling activation.

**Conclusions:**

Our results showed that the *Treponema pallidum* Tp0136 protein stimulated human platelet activation and aggregation by downregulating PAR1 and triggered PAR1-dependent G_q_ and G_i_ pathway activation. These findings may contribute to our understanding of the self-healing of chancroid in early syphilis.

## Highlights

1. Tp0136 enhances platelet activation and aggregation by downregulating PAR1.
2. Tp0136-downregulated PAR1 selectively stimulates PAR1-dependent G_q_ and G_i_ pathway activation.

## Introduction

Syphilis, a chronic multistage disease punctuated by asymptomatic periods of latency, is caused by the spirochete *Treponema pallidum* subsp. *pallidum* (hereafter *T*. *pallidum*) and is primarily transmitted sexually or vertically during pregnancy (1). Syphilis is clinically manifested when spirochetes replicating at the site of inoculation induce a local inflammatory response sufficient to generate a papule that subsequently ulcerates, forming a chancre; chancres are the defining lesions of primary syphilis and are typically painless and resolve spontaneously (2). Thus far, our understanding of the mechanism of chancre self-healing in syphilis is limited.

Wound healing is one of the most complex processes in the human body, involving the spatiotemporal synchronization of multiple cell types with different roles in the phases of hemostasis, inflammation, growth, reepithelialization, and remodeling (3). The first reaction to a wound is constriction of the injured blood vessels and activation of platelets that adhere to the damaged site and aggregate to form a fibrin clot, resulting in the early stabilization of platelet thrombi that thereby initiate hemostasis (4, 5). Human platelets express protease-activated receptor 1 (PAR1), the prototypical member of the G-protein-coupled receptor family, which is activated by a variety of proteases (6). The activation of PAR1 is sufficient to trigger platelet secretion and aggregation (7), and PAR1 can couple with members of the G_q_, G_12/13_, and G_i_ families to impact a substantial of signaling pathway networks (8).

Tp0136, an outer membrane lipoprotein of *T*. *pallidum*, is also an adhesin that is predicted to bind to different host cells and thereby mediate the colonization of *T*. *pallidum* in different tissues during infection (9, 10). Our previous study found that Tp0136 promoted the migration and proliferation of fibroblasts (11) and microvascular endothelial cells (12), which could contribute to the mechanism of chancre self-healing in syphilis. In addition, high titers of anti-Tp0136 antibodies promoted the infiltration of inflammatory cells into local lesions and intensified tissue damage, thus delaying wound healing (13). While platelets have been shown to be a *T*. *pallidum* target (14), whether *T*. *pallidum* activates platelets through Tp0136 and promotes platelet aggregation to mediate the self-healing of chancre remains unclear. In the current study, we performed a series of *in vitro* experiments to elucidate the effect of Tp0136 on platelet activation and aggregation and analyzed PAR1 receptors and subsequent signaling pathways that involved in this process.

## Materials and Methods

### Preparation of the Tp0136 Protein and Removed Endotoxin

Full-length Tp0136 was directly cloned into the pEXP-5-CT vector, and the Tp0136-His-Tag protein was purified by affinity chromatography using Ni-NTA as described previously (12). Endotoxin was removed from the recombinant Tp0136 protein with an EtEraser™ Endotoxin Removal Kit (Chinese Horseshoe Crab Reagent Manufactory, Ltd., Xiamen, China). Tachypleus amebocyte lysate (Chinese Horseshoe Crab Reagent Manufactory, Ltd., Xiamen, China) was used to detect endotoxin in the Tp0136 preparation, which was found to have less than 0.05 endotoxin units (EUs)/mL. A cytotoxicity assay was performed to evaluate the effect of Tp0136 on endothelial cells viability using a lactate dehydrogenase kit (NEOBIOSCIENCE Biotechnology Co., Ltd. Beijing, China). The results showed no significant cytotoxicity in Tp0136-treated cells.

### Preparation of Human Platelets

Platelets from healthy volunteers were separated by the differential centrifugation of whole blood in anticoagulation tubes containing 3.8% sodium citrate as previously described (15, 16). Platelet-rich plasma (PRP) was separated from the plasma samples and centrifuged at 180 g for 15 minutes at room temperature. The samples were then centrifuged at 800 g for 20 minutes to obtain the platelet precipitates, which were then resuspended in modified Tyrode’s solution (Solarbio, Beijing, China) and diluted to 2.0-3.0 × 10^8^/mL for the following experiments. The studies involving human participants were reviewed and approved by the Ethics Committee of Zhongshan Hospital, Xiamen University. All volunteers provided written informed consent in accordance with the Declaration of Helsinki.

### Assessment of Platelet Adhesion and Spreading

Platelet adhesion assays were performed according to Boncler et al. (15). Samples in 96-well microplates coated with 2 mg/mL fibrinogen (4°C, overnight) were blocked with 0.2% bovine serum albumin (1 hour, 37°C). The PRP samples were incubated for 15 minutes with thrombin (0.5 U/mL), Tp0136 (10 μg/mL), Tp17 (10 μg/mL) or phosphate-buffered saline (PBS) in the absence or presence of different antagonists or agonists. Then, 50 μL aliquots of PRP were added to the wells for 1 hour at 37°C. After washing, the wells were filled with a substrate solution and incubated for 1 hour. To estimate “total platelet adhesion”, the PRP samples were mixed with the substrate solution and added to the uncoated wells. Platelet-deficient plasma was used as a blank control. Sodium hydroxide (2 M) was added to stop the enzymatic reaction, and the absorbance at 405 nm was read using a microplate analyzer (Thermo Scientific Multiskan FC, USA). The percentage of adherent platelets was calculated using the following formula: (sample–blank)/(total–blank) × 100.

Platelet cytoskeleton staining assays were performed as described in a previous study (17). PRP samples were treated with thrombin (0.5 U/mL), Tp0136 (10 μg/mL), Tp17 (10 μg/mL) or PBS with or without RWJ56110 (a PAR1 antagonist) (1 μM) or an anti-PAR1 antibody (1:100) and placed onto fibrinogen-coated Millicell glass slides for 1 hour at 37°C. Adherent platelets were fixed with 4% paraformaldehyde, permeabilized with 0.1% Triton X-100, blocked with 5% bovine serum albumin and then stained with TRITC-labeled phalloidin at room temperature for 30 minutes. Fluorescence images were obtained on a confocal microscope (Zeiss Axio Observer LSM780, Oberkochen, Germany). The number of platelet adhesion events and the platelet spreading surface area were determined using NIH ImageJ software (NIH, Bethesda, MD, USA).

### Determination of Platelet Aggregation

Agonist-induced platelet aggregation was measured using the PL-16 aggregometer (Sinnowa, Jiangsu, China) at 37°C and a constant stirring speed of 50 g to analyze platelet function. Briefly, platelets were incubated with thrombin (0.5 U/mL), Tp0136 (10 μg/mL), Tp17 (10 μg/mL) or PBS with or without different inhibitors or antagonists (RWJ56110, U73122 or PTX) for 5 minutes at 37°C and assessed on an aggregometer according to the manufacturer’s protocol.

### Platelet-Mediated Clot Retraction Assay

The platelet clot retraction experiment was performed as described by Ren et al. (17). After incubation with 10 μg/mL Tp0136 and Tp17 (PBS as a control), platelets were stimulated with 20 μg/mL fibrinogen and 0.5 U/mL thrombin and recorded at the indicated time point using a camera. The clot area was quantified based on the ratio of the clot area to the platelet suspension area at different time points using ImageJ software (National Institute of Mental Health, Bethesda, MD, USA).

### Platelet Activation Assays

Measurement of the platelet surface molecules P-selectin (18, 19) and PF4 (20) as indices of platelet activation was performed by flow cytometry (BD FACSCanto II, NJ, USA) and the enzyme-linked immunosorbent assay (ELISA). For the P-selectin measurement, platelets were diluted to 2.0 × 10^8^/mL with modified Tyrode’s buffer and incubated for 15 minutes with thrombin (0.5 U/mL), Tp0136 (10 μg/mL) or PBS in the absence or presence of different antagonists or agonists. The aliquots (100 μL) were then stained with an APC-labeled anti-human CD41 antibody (Biolegend, Shanghai, China) as a platelet identifier and with a FITC-labeled anti-human P-selectin antibody (Biolegend, Shanghai, China) before being analyzed by flow cytometry. Data were analyzed with FlowJo (TreeStar Software, Ashland, OR, USA). The levels of PF4 in PRP samples were assessed by ELISA (Human PF4 Simple Step ELISA^®^ Kit, Abcam, MA, USA).

### cAMP Release Assays

Cyclic adenosine monophosphate (cAMP) in PRP samples was assessed by a competition-based assay (cAMP ELISA Detection Kit, GenScript, NJ, USA). The PRP samples were preincubated with iloprost (final concentration, 100 ng/mL) for 2 minutes, after which 10 μg/mL Tp0136 (PBS as a control) and RWJ56110 (1 μM) were added alone or in combination. The samples were incubated for 15 minutes at 37°C and then analyzed for cAMP content.

### Determination of Ca^2+^ Fluxes

The kinetics of intracellular Ca^2+^ mobilization were assessed as previously described (21). Platelets diluted in modified Tyrode’s buffer (2.0 × 10^8^/mL) were incubated in Fluo-3-AM solution (Sigma, MO, USA) for 30 minutes at 37°C. After determining the basal Ca^2+^ levels, Tp0136 (10 μg/mL) was added to the tube in the absence or presence of RWJ56110 (1 μM), and the samples were assayed immediately. Flow cytometric analysis was then performed (BD FACSCanto II, NJ, USA).

### Cell Culture, Plasmid Cloning and Transfection

Chinese hamster ovary (CHO) cells were cultured in DMEM supplemented with fetal bovine serum (10% vol/vol), penicillin (100 U/mL) and streptomycin (100 μg/mL). Full-length human-PAR1 cDNA was amplified and cloned into a pcDEF3-CMV-T7-tagged vector (MiaoLingBio, Wuhan, China) to obtain a pcDEF3/PAR1 T7-tagged wild-type plasmid that was used to generate all mutants. pcDEF3 vectors encoding the T7-tagged PAR1 mutants L38S, D39S, P40N, R41A, S42D, and F43R were generated as described previously (22). CHO cells were transiently transfected with pcDEF3/PAR1 T7-tagged wild-type or PAR1 mutants using Lipofectamine™ 3000 Reagent (Invitrogen, Carlsbad, CA, USA) according to the manufacturer’s recommendations and assessed by flow cytometry.

### Western Blotting Assays

Platelets were stimulated with Tp0136 at different concentrations, and the PAR1 protein levels were measured by western blot as described previously (22). Platelets were treated with thrombin receptor activating peptide (TRAP)-6 (10 μM) or Tp0136 (10 μg/mL) in the absence or presence of RWJ56110 (1 μM) for 15 minutes at 37°C. The cell lysates were collected, and the protein levels of phosphorylated and total PI3K, Akt, and p38 were detected by western blotting (23). Antibodies against PAR1 and PI3K/Akt/p38 signaling pathway components were purchased from Cell Signaling Technology (Danvers, MA, USA) or R&D Systems (Minneapolis, MN, USA).

### Data Analysis and Statistics

All results are expressed as the mean ± standard error of the mean (SEM). Multiple groups were compared by one-way analysis of variance (ANOVA). Comparisons between two groups were made using paired Student’s t-tests. Differences in calcium levels over time were assessed by repeated-measures ANOVA followed by Dunnett’s *post hoc* test. All calculations were performed with the GraphPad Prism 6.0 program (version 5.0, GraphPad Software Inc, San Diego, CA, USA) and IBM SPSS statistics version 26 (SPSS, Inc., Chicago, IL, USA). A two-tailed *P* value of <0.05 was considered statistically significant.

## Results

### Tp0136 Promoted Human Platelet Adhesion and Aggregation

**Figure 1:**
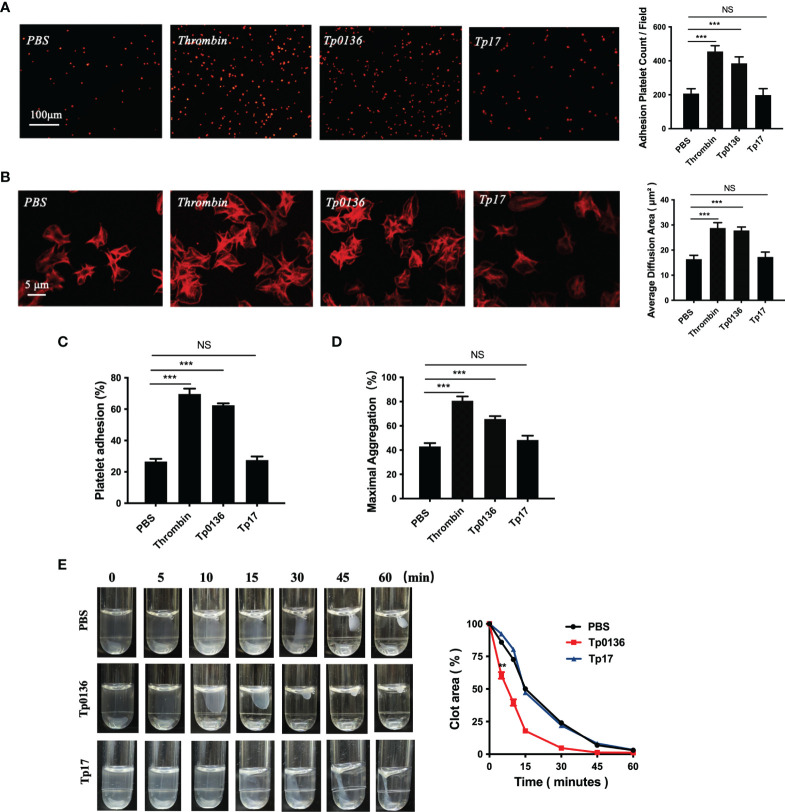
Tp0136 promoted human platelet adhesion and aggregation. **(A, B)** Effect of Tp0136 on platelet adhesion as determined by staining with TRITC-labeled phalloidin. Statistical data were determined based on the number of platelet adhesions **(A)** and were calculated from the mean of the average surface area of individual platelets **(B)**. **(C)** Platelet adhesion as assessed by ELISA. **(D)** The maximum aggregation rate of platelets was determined by an aggregometer. **(E)** Platelet clot retraction was quantified by the ratio of the clot area to the platelet suspension area at different time points. The values are presented as the mean ± SEM of experimental triplicates and are representative of the results of three independent experiments. Values among multiple groups were compared by one-way ANOVA. Comparisons between two groups in the platelet clot retraction experiment were made using a paired t-test (NS, no significance, **P < 0.01, ***P < 0.001).

To elucidate the effect of Tp0136 on platelet adhesion and aggregation, platelets were treated with the Tp0136 protein, Tp17 protein, PBS (as a blank control) and thrombin (as a positive control). Platelet staining with TRITC-phalloidin revealed that the number of platelets adhered to the precoated fibrinogen in the Tp0136 group was higher than that in the PBS group (*P <*0.001), Tp17 group was no change (**Figure 1A**). The platelet spreading surface area was assessed using NIH ImageJ software, and the average diffusion area of Tp0136-treated platelets immobilized on fibrinogen was markedly increased compared with that of PBS-treated platelets (*P <*0.001), and Tp17-treated platelets was no statistical difference (**Figure 1B**). The effect of Tp0136 on platelet adhesion to fibrinogen was confirmed by the significantly higher adhesion of Tp0136-stimulated platelets to the fibrinogen-coated surface as determined by ELISA (*P <*0.001), compared with that of PBS-treated platelets, and platelet adhesion was no change stimulated by Tp17 (**Figure 1C**). The platelet aggregation assay (**Figure 1D**) revealed an increased response to Tp0136-treated platelets (*P <*0.001) and no increased in the Tp17-treated platelets. In addition, the result of the clot retraction assay showed that Tp0136-treated human platelets accelerated platelet aggregation and that the aggregation area was significantly lower than that in the PBS group after 5 minutes (*P <*0.01). The Tp0136-treated human platelets almost formed a very small clot after 30 minutes, while these was no difference between Tp17-treated group and PBS group, as demonstrated in **Figure 1E**. These data indicated that Tp0136 promoted human platelet adhesion and aggregation.

### Tp0136 Promoted Human Platelet Activation

**Figure 2:**
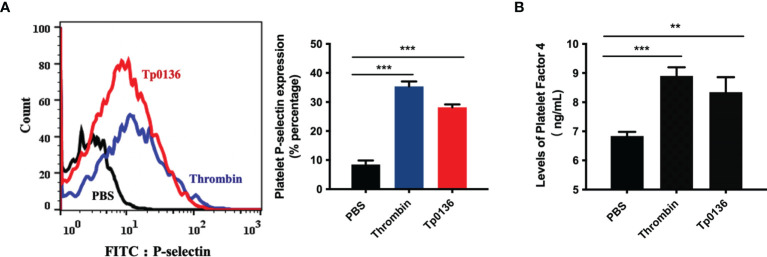
Tp0136 promoted human platelet activation. **(A)** Expression of platelet P-selectin as measured by flow cytometry. A representative histogram is shown. Statistical data were analyzed using the X geometric mean fluorescence (left) and the percentage of gated cells (right). **(B)** PF4 expression as determined by ELISA. The values are presented as the mean ± SEM of experimental triplicates and are representative of the results of three independent experiments. Values among multiple groups were compared by one-way ANOVA (**P < 0.01, ***P < 0.001).

Given the promotional effect of Tp0136 on platelet adhesion and aggregation, we next investigated whether Tp0136 affects platelet activation. The flow cytometry results showed that the surface expression of P-selectin was increased in the Tp0136-treated platelet group by threefold compared with that in the PBS group (*P <*0.001) (**Figure 2A**). In addition, Tp0136 significantly promoted the secretion of PF4 (vs. PBS-treated group, *P <*0.01) (**Figure 2B**). These data indicated that Tp0136 promoted platelet activation.

### Tp0136 Promoted Platelet Activation and Aggregation Through PAR1

**Figure 3:**
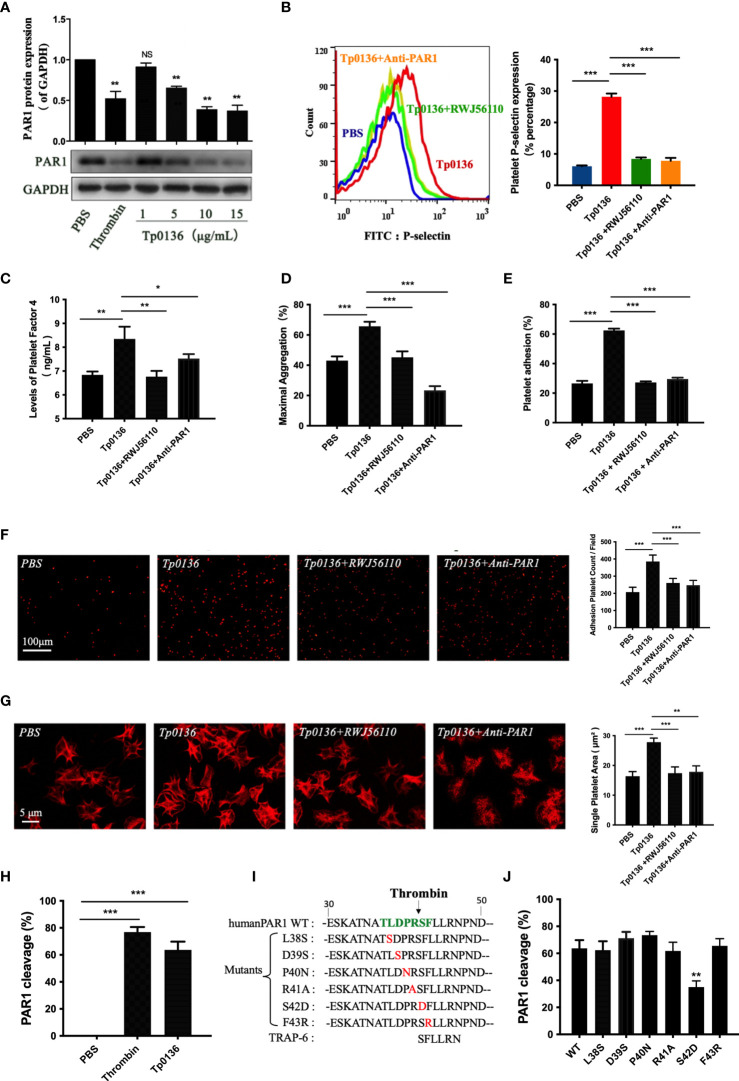
Tp0136 promoted platelet activation and aggregation through PAR1. **(A)** PAR1 protein expression as determined by western blotting. **(B-G)** Effects of the PAR1 antagonist RWJ56110 and the anti-PAR1 antibody on platelet activation and aggregation induced by Tp0136. **(B)** P-selectin expression as measured by flow cytometry. **(C)** PF4 expression as measured by ELISA. **(D)** Maximal platelet aggregation as determined by an aggregometer. **(E)** Platelet adhesion as assessed by ELISA. **(F,G)** Platelet adhesion as assessed by staining with TRITC-labeled phalloidin. Statistical data are based on the number of platelet adhesions **(F)** and were calculated from the mean of the average surface area of individual platelets **(G)**. **(H)** Effect of Tp0136 on PAR1 as determined by flow cytometry. **(I)** Amino acid sequences of WT (wild-type) PAR1, PAR1 proteins with mutations in the extracellular domain and a PAR1 peptide agonist (TRAP-6). **(J)** Exploration of the Tp0136 protein sites that act on PAR1. The values are presented as the mean ± SEM of experimental triplicates and are representative of the results of three independent experiments. Values among multiple groups were compared by one-way ANOVA. Comparisons between two groups were made using a paired t-test (NS, no significance, *P < 0.05, **P < 0.01, ***P < 0.001).

Human platelets express PAR1, and the activation of PAR1 is sufficient to trigger platelet secretion and aggregation (7). To confirm the regulation of platelet PAR1 receptors by Tp0136, platelets were treated with Tp0136 at different concentrations. The protein expression of PAR1 was decreased after treatment with Tp0136 at a concentration of 5 μg/mL (*P <*0.01), and the best response was achieved with 15 μg/mL Tp0136 (*P <*0.01) (**Figure 3A**), indicating a concentration-dependent pattern. In addition, the pretreatment of platelets with RWJ56110 (a specific PAR1 antagonist) or an anti-human-PAR1 antibody ameliorated the activation and aggregation of platelets induced by Tp0136. RWJ56110 and the human-PAR1 antibody significantly reduced the expression of P-selectin on platelets (*P <*0.001) (**Figure 3B**), the secretion of PF4 (*P <*0.05) (**Figure 3C**), platelet aggregation (*P*<0.001) (**Figure 3D**), platelet adhesion (*P <*0.001) (**Figures 3E, F**) and the average platelet diffusion area (*P <*0.01) (**Figure 3G**). Taken together, these results demonstrate that PAR1 is essential for the Tp0136-mediated promotion of platelet activation and aggregation.

Serine proteases, such as plasmin, thrombin, and activated protein C, hydrolyze PAR1 to generate a tethered ligand that, in turn, activates PAR1 by interacting with the body of the receptor, thus triggering transmembrane signaling (7, 24, 25). In our study, the cleavage of platelet PAR1 by Tp0136 was confirmed in CHO cells expressing the T7-tagged wild-type PAR1 plasmid (**Figure 3H**). To identify the specific cleavage site of Tp0136, we performed site-directed mutations of the key residues of the PAR1 N-terminus between amino acids 38 and 43, generating the T7-tagged PAR1 mutants L38S, D39S, P40N, R41A, S42D, and F43R (**Figure 3I**), and expressed them in CHO cells. The cleavage of PAR1 by Tp0136 was suppressed in T7-S42D PAR1-transfected cells compared with T7-wild-type PAR1-transfected cells (**Figure 3J**). Thus, Tp0136 cleaves PAR1 at LDPR^41^↓S^42^FL to generate the S^42^FLLRN-tethered ligand (TRAP-6), similar to that produced by thrombin (22).

### Tp0136 Enhanced G_q_ Signaling Through PAR1 During Platelet Activation

**Figure 4:**
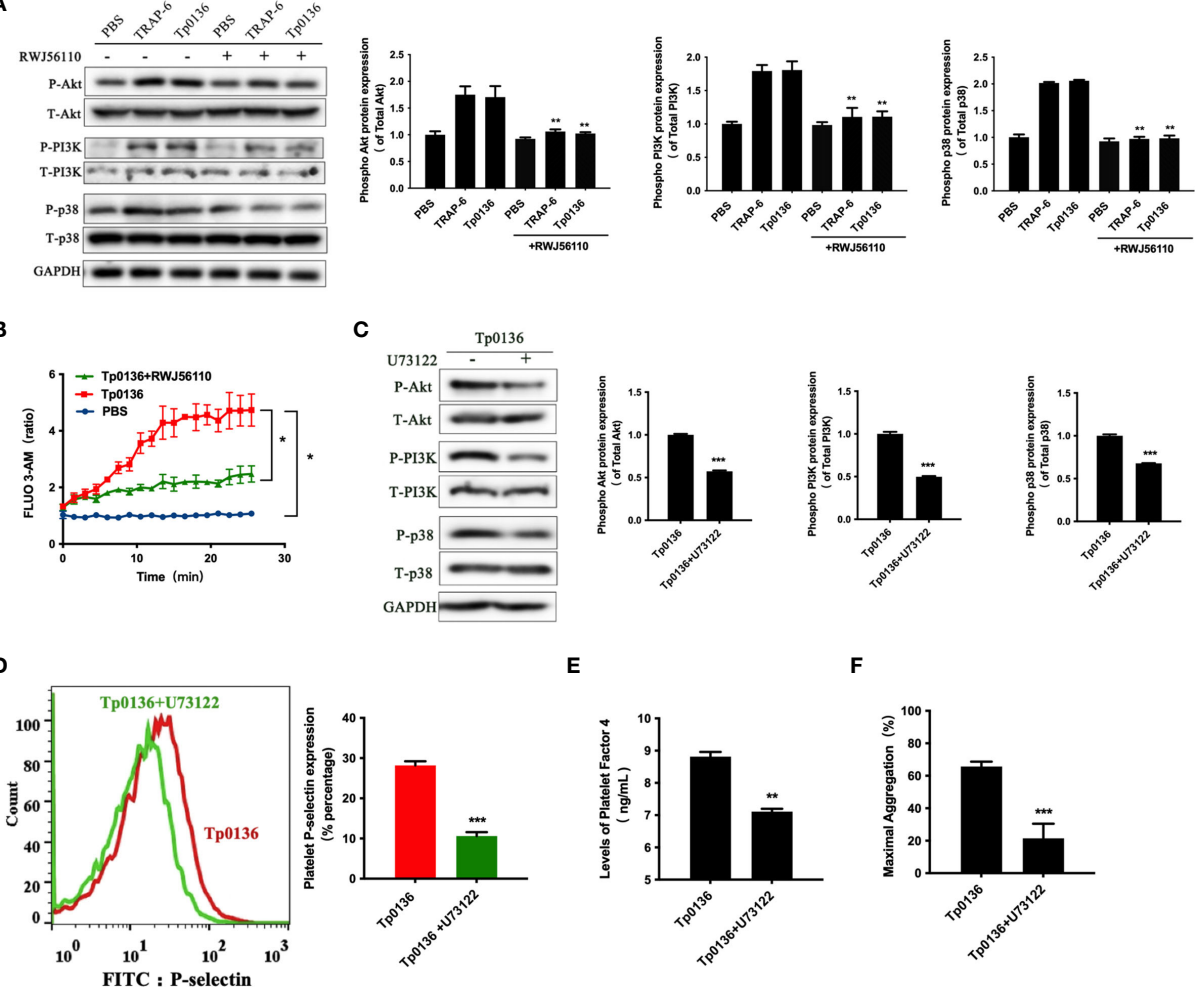
Tp0136 enhanced G_q_ signaling through PAR1 during platelet activation. **(A)** The protein expression of phosphorylated/total p38/Akt/PI3K and GAPDH was assessed by western blotting. **(B)** Cytosolic free Ca^2+^ was measured by flow cytometry. **(C)** The protein expression of phosphorylated/total p38/Akt/PI3K and GAPDH was assessed by western blotting. **(D)** Expression of platelet P-selectin as measured by flow cytometry. A representative histogram is shown. **(E)** PF4 as determined by ELISA. **(F)** Maximal platelet aggregation as determined by an aggregometer. The values are presented as the mean ± SEM of experimental triplicates and are representative of the results of three independent experiments. Comparisons between the two groups were analyzed using a paired t-test. Differences in the calcium levels over time were determined by repeated-measures ANOVA followed by Dunnett’s *post hoc* test (*P < 0.05, **P < 0.01, ***P < 0.001).

Activated PAR1 can couple with members of the G_q_ and G_i_ families and trigger numerous intracellular signaling pathways (8). To determine whether Tp0136 activated G_q_-protein-coupled pathways through PAR1 to promote platelet activation and aggregation, the expression levels of Akt, PI3K and P38 were detected. As shown in **Figure 4A**, Tp0136 triggered the phosphorylation of Akt (*P <*0.01), indicating PI3K activation (*P <*0.01), and the phosphorylation of p38 (*P <*0.01), confirming G_q_-dependent signaling, similar to TRAP-6. To determine whether Tp0136 enhances G_q_ signaling through PAR1, the PAR1 antagonist RWJ56110 was utilized. Interestingly, the RWJ56110 treatment of platelets significantly reduced the activation ability of Akt, PI3K and p38 compared with that of the conditioned media-treated controls, reversing the changes caused by Tp0136 (**Figure 4A**). In addition, Tp0136 significantly elicited a G_q_-triggered increase in intraplatelet calcium levels (*P <*0.05), as measured using the Fluo-3AM calcium indicator, and this effect was inhibited by the PAR1 antagonist RWJ56110 (*P <*0.05) (**Figure 4B**). The involvement of G^q^-dependent signaling during Tp0136-induced platelet activation was significantly inhibited by U73122, a phospholipase C inhibitor (*P <*0.001) (**Figure 4C**). The inhibitor U73122 significantly abolished the potentiating effect of Tp0136 on P-selectin expression (*P <*0.001) (**Figure 4D**), the secretion of PF4 (*P <*0.01) (**Figure 4E**) and platelet aggregation (*P <*0.001) (**Figure 4F**).

### Tp0136 Enhanced G_i_ Signaling Through PAR1 During Platelet Activation

**Figure 5:**
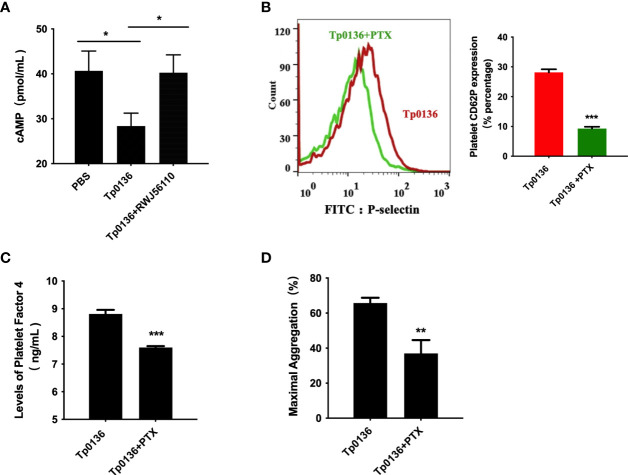
Tp0136 enhanced G_i_ signaling through PAR1 during platelet activation. **(A)** cAMP as analyzed by ELISA. **(B)** P-selectin as measured by flow cytometry. A representative histogram is shown. **(C)** PF4 as measured by ELISA. **(D)** Maximal platelet aggregation as determined by an aggregometer. The values are presented as the mean ± SEM of experimental triplicates and are representative of the results of three independent experiments. Comparisons between the two groups were analyzed using a paired t-test (*P < 0.05, **P < 0.01, ***P < 0.001).

Furthermore, cAMP was measured to determine whether the effects of Tp0136 on platelets involve G_i_-dependent signaling pathways. The iloprost-induced increase in the intraplatelet cAMP concentration was affected by Tp0136 (*P <*0.05), and pretreatment with RWJ56110 increased the cAMP level compared to that in the group stimulated with only Tp0136 (*P <*0.05) (**Figure 5A**). To assess the contribution of G_i_ signaling to the activation of platelets by Tp0136, pertussis toxin (PTX), an inhibitor of the G_i_ signaling pathway, was utilized to specifically inhibit G_i_. PTX significantly reversed the enhancing effect of Tp0136 on platelet surface P-selectin expression (*P <*0.001) (**Figure 5B**) and PF4 granular secretion (*P <*0.01) (**Figure 5C**) and weakened the enhancement of platelet aggregation induced by Tp0136 (*P <*0.01) (**Figure 5D**), showing that G_i_-dependent signaling was involved in the activating effect of Tp0136 on platelets.

## Discussion

The well-recognized capacity of *T. pallidum*, the etiological agent of venereal syphilis, for early dissemination and immune evasion has earned it the designation of ‘the stealth pathogen’ (26). Patients with primary syphilis present with typical chancres, painless ulcerations, that heal spontaneously over several weeks, which gives the illusion that the person has not been infected with syphilis and thus causes the best treatment period to be missed; thereafter, *T. pallidum* enters a latent state, inducing an insidious infection. Self-healing of chancre is essentially wound healing, which is an important multifaceted and complicated process in humans and animals that is governed by sequential but overlapping phases, including the hemostatic, inflammatory, proliferative, and remodeling phases (27). It is important to understand the first signals that activate the cellular response of injured tissue. After injury to the skin, the exposed subendothelial, collagen and tissue factors activate platelet aggregation, which results in degranulation and the release of chemotactic and growth factors to form the clot that initiates wound healing (28). In the present study, we found that Tp0136, a predicted *T. pallidum* adhesin that mediates its colonization during infection (9), enhanced platelet activation and aggregation through PAR1 and then initiated the receptor G_q_ and G_i_ signaling pathways. This process may represent the first step of platelet recruitment to syphilitic lesion sites and the initiation of wound healing. However, a recently research by Cameron group reported that *T. pallidum* directly, preferentially, and reversibly interacted with platelets, altered their movement and increased blood-brain barrier permeability, eventually facilitating their dissemination (14). Tp0136 as an important adhesion protein of *T. pallidum*, here we found that it could stimulate platelet activation and aggregation. Therefore, Tp0136 protein could play an important role in interaction with platelet mediating the dissemination of *T. pallidum*, which would need more research.

In our study, Tp0136 activated platelets and promoted platelet-fibrinogen adhesion and aggregation, resulting in the formation of platelet clots, which are naturally involved in wound healing (29). Upon activation, platelets secrete more than 300 active substances from their intracellular particles. Herein, Tp0136 activated platelets and promoted the platelet secretion of granules, such as PF4, P-selectin, and Ca^2+^, into the surrounding cellular milieu. These secreted platelet granule components contribute to blood coagulation (30). P-selectin is an inflammatory coagulation biomarker involved in clotting (31). Our results showed that Tp0136 promoted the expression of P-selectin in platelets and promoted platelet activation and aggregation, which may have initiated the self-healing of chancres in the early stages of syphilis. Of course, this phenomenon needs to be further studied in animals (*in vivo*) infected with syphilis.

PARs are G-protein-coupled receptors that utilize a fascinating mechanism to convert an extracellular proteolytic cleavage event into a transmembrane signal; these receptors carry their own ligands, which remain hidden until unmasked by receptor cleavage (8). PAR1, the prototype of this family, is activated when thrombin cleaves its amino-terminal extracellular domain at a specific site. This cleavage reveals a new N-terminus that acts as a tethered ligand for intramolecular binding to the body of the receptor and thus affects transmembrane signaling (32). We observed that Tp0136 enhanced platelet activation and aggregation through PAR1. We next carried out site-directed mutagenesis of PAR1 and found that Tp0136 acted on the N-terminal extracellular domain of PAR1 between residues 41 and 42 (LDPR^41^↓S^42^FL), which was consistent with thrombin. However, whether the Tp0136 protein has the characteristics of an active enzyme still needs to be determined.

PAR1 can couple to members of the G_q_ and G_i_ families and thus to a host of intracellular effectors. Our results showed that Tp0136 enhanced platelet activation through PAR1, thereby initiating G_q_- and G_i_- activated intracellular pathways and thus predisposing platelets to be fully activated by a subsequent subthreshold stimulus. G_q_ generates a pathway for calcium-regulated kinases, mitogen-activated protein kinase cassettes, and other proteins that mediate cellular responses ranging from particle formation to integrin activation to platelet aggregation (33, 34). Furthermore, Akt is a serine/threonine-specific protein kinase that plays a key role in platelet aggregation, integrin signaling, particle secretion, and clot retraction (35). In our study, Tp0136 stimulated G_q_ activation through PAR1 in human platelets, as shown by Akt-PI3K activation and p38 phosphorylation, and increased the release of intraplatelet Ca^2+^, a key second messenger, from intracellular stores (36, 37). As reported herein and in agreement with the findings of others (38), the treatment of platelets with U73122, a phospholipase C inhibitor, partially attenuated the upregulated expression of P-selectin and PF4 as well as the subsequent platelet adhesion and aggregation. These observations clearly underscore the critical regulatory effect of Tp0136 on the G_q_- activated intracellular pathways downstream of PAR1. In addition, Tp0136 reduced the cAMP levels in iloprost-exposed platelets. Cytosolic cAMP is synthesized by adenylyl cyclase and is known as a powerful inhibitor of platelet aggregation (39). Moreover, PTX, which inhibits G_i_-receptor coupling pathways, affected the potentiating activity of Tp0136 on platelet aggregation. Based on the information discussed above, Tp0136 induces Akt-PI3K activation, p38 phosphorylation and Ca^2+^ movement in platelets; however, to achieve full platelet activation, Tp0136 must be induced by regulating the intracellular cAMP levels and thereby triggering the concomitant G_i_ signaling pathway. A similar mechanism has been reported for other agents enhancing platelet activation, such as PGE_2_ (24) and MMP-2 (22), and explains the difference between platelet primers and full platelet agonists (40).

Several limitations should be noted. First, we showed that Tp0136 activated platelets to promote their aggregation *in vitro*, and further studies, such as *in vivo* experiments, is needed to confirm our *in vitro* findings. Second, we detected changes in the protein expression of only PAR1 and related downstream signaling molecules, and further study is needed to determine whether other platelet receptors are involved in this process. Third, the effect of Tp0136 promoting platelet activation and aggregation on the development of syphilis infection remains to be further studied.

**Figure 6:**
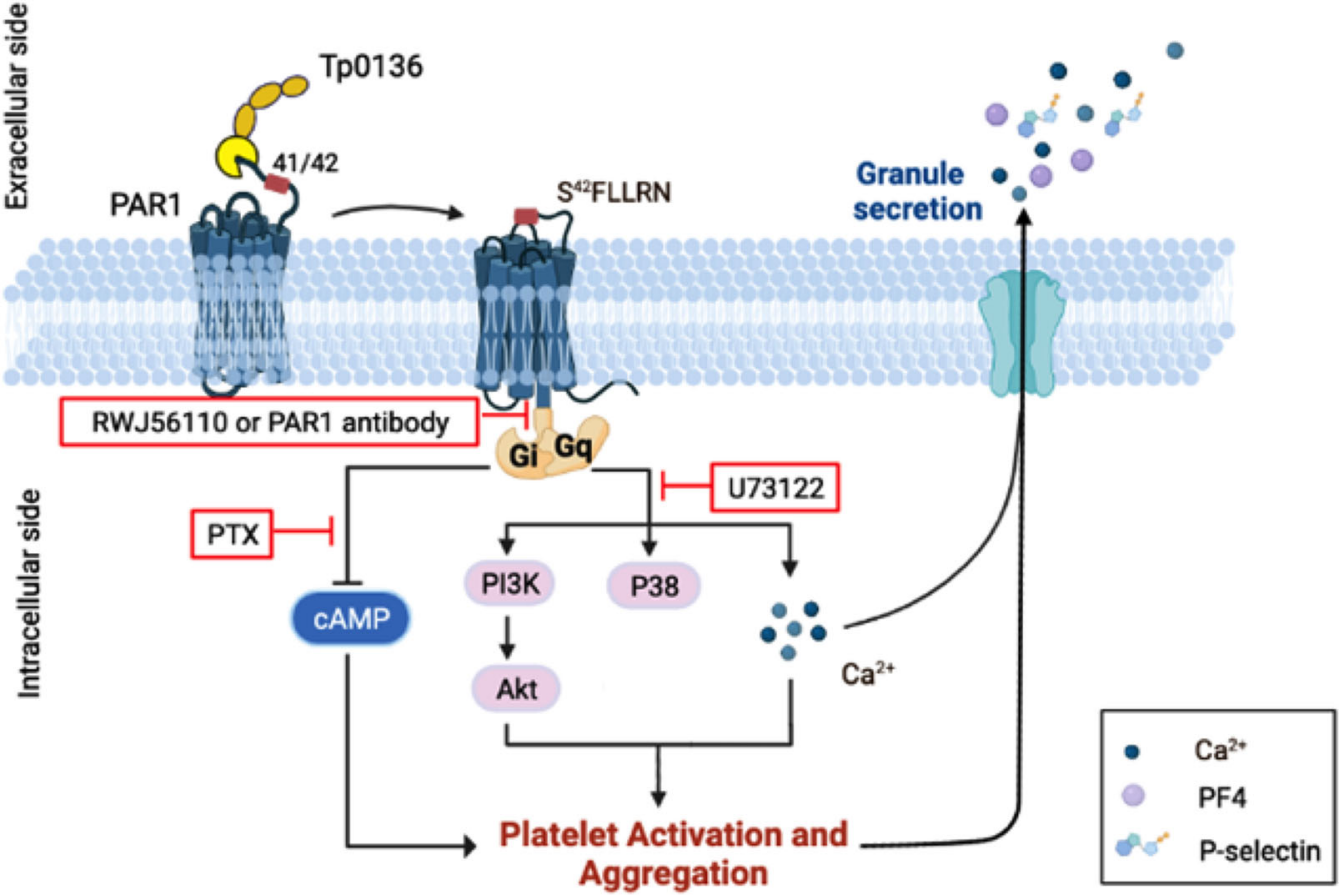
Schematic model of the mechanism by which Tp0136 modulates platelet signaling. Tp0136 promotes human platelet activation and aggregation through PAR1 and generates an S^42^FLLRN-tethered ligand that interacts with the receptor to induce PAR1 signaling. In particular, Tp0136 activates the G^q^ signaling pathway through PAR1, thereby inducing PAR1-dependent Akt-PI3K activation, p38 phosphorylation and Ca^2+^ flux, and the G^i^ pathway, thereby attenuating the release of cytosolic cAMP.

In conclusion, were herein elucidated a mechanism of platelet activation and aggregation in which Tp0136 effects platelets through PAR1 and thereby triggers downstream G_i_ and G_q_ signaling (**Figure 6**). This study suggests that Tp0136 plays a role in platelet function, and the elucidation of relevant mechanisms represents another step toward understanding chancre self-healing in the early stages of syphilis.

## Data Availability Statement

The original contributions presented in the study are included in the article/supplementary material. Further inquiries can be directed to the corresponding author.

## Ethics Statement

The studies involving human participants were reviewed and approved by the Ethics Committee of Zhongshan Hospital, Xiamen University. All volunteers provided written informed consent in accordance with the Declaration of Helsinki.

## Author Contributions

QY-X was first author. TC-Y was corresponding author. TC-Y and QY-X designed the study and drafted the manuscript, TC-Y and LR-L critical review and revision of the manuscript. QY-X and YJ-W performed experiments. LL-L was responsible for statistical analysis and validation. All authors agree to be accountable for the content of the work. All authors read and approved the final manuscript.

## Funding

This work was supported by the National Natural Science Foundation of China (grant numbers 82172331, 81972028, 81973104, 81971147, 81772260, 81771312), the Key Projects for Province Science and Technology Program of Fujian Province, China (grant number 2020D017) and the Natural Science Foundation of Fujian Province, China (grant number 2021J02055). The funders played no role in the study design, data collection, or analyses, the decision to publish, or manuscript preparation.

## Conflict of Interest

The authors declare that the research was conducted in the absence of any commercial or financial relationships that could be construed as a potential conflict of interest.

## Publisher’s Note

All claims expressed in this article are solely those of the authors and do not necessarily represent those of their affiliated organizations, or those of the publisher, the editors and the reviewers. Any product that may be evaluated in this article, or claim that may be made by its manufacturer, is not guaranteed or endorsed by the publisher.
